# Generic versus brand-name over-the-counter analgesics: knowledge and attitudes among Swedish pharmacy customers

**DOI:** 10.1186/s40545-020-00269-5

**Published:** 2020-10-01

**Authors:** Helle Håkonsen, Maria Wängberg, Dina Alani, Tove Hedenrud

**Affiliations:** School of Public Health and Community Medicine, Institute of Medicine, University of Gothenburg, P.O. Box 453, 405 30 Gothenburg, SE Sweden

## Abstract

**Background:**

Due to a liberalisation reform in 2009, the availability of over-the-counter (OTC) analgesics has increased significantly in the Swedish market over the past decade. With the increasing number of generic products available on the market and the possibility of buying OTC drugs from non-pharmacy outlets, a key to safe drug use is that consumers possess the necessary knowledge to differentiate between the different brands and choose the appropriate drug for their ailments. The aim of this study was to investigate Swedish consumers’ knowledge of and attitudes towards generic OTC analgesics.

**Methods:**

A sample of 209 Swedish adults (66% women; mean age 43.1 years) who bought OTC analgesics at a community pharmacy in one of the country’s three largest cities responded to a structured questionnaire. The questions related to knowledge of active substances, the use and choice of OTC analgesics (generic or original brand), attitudes towards generic OTC analgesics, information received about OTC analgesics and experience with generic substitution of prescription drugs.

**Results:**

Almost one in five reported weekly use of OTC analgesics, and 32% assigned minimum three out of four active substances to the correct brand(s) of OTC analgesics. Among the 50 participants (24%) who assigned all four active substances correctly, it was predominantly women and participants with higher education. Four out of five participants were positive towards the cheaper brands, and 69% reportedly chose cheaper generic brands over more expensive brands. Knowledge about the active substances of different brands was associated with positive attitudes towards generic products.

**Conclusion:**

Swedish pharmacy customers have to a varying extent the knowledge to differentiate between different brands of commonly used OTC analgesics in terms of active substances. There is a predominantly positive attitude towards generic OTC analgesics, although some consider generic drugs to be inferior and stay loyal to the original brands.

## Background

A decade ago, the Swedish pharmacy monopoly was lifted, and a selection of over-the-counter (OTC) drugs was permitted for sale by non-pharmacy outlets. The intention was to increase the availability of OTC drugs and stimulate price competition between different suppliers [1]. In general, increased availability of OTC drugs may provide the possibility for people to take more responsibility of the management of their own health and enhance self-care of minor ailments. Furthermore, responsible and appropriate self-medication with OTC drugs can provide significant economic benefits for society [2]. However, responsible self-medication depends on people’s ability to choose an appropriate drug for their ailments and to use the drug correctly.

Although community pharmacies have remained the preferred retailer for OTC drugs among Swedes, a majority of the population have sometimes obtained OTC drugs from a non-pharmacy outlet since these drugs became available on the larger market [3]. Analgesics, which constitute the most commonly used group of OTC drugs, is the group of drugs most frequently bought from non-pharmacy outlets [3]. These drugs are generally well known and perceived by consumers as less risky than other OTC drugs [4]. However, there is potential for serious adverse events, even in therapeutic doses, for individuals with certain conditions. For example, intake of nonsteroidal anti-inflammatory drugs (NSAIDs) is associated with various adverse events, including gastrointestinal, renal and cardiovascular complications [5]. Paracetamol is especially associated with the risk of hepatic toxicity in large doses [6] and also with the adverse events that often are observed with NSAIDs at standard analgesic doses [7]. In Sweden, a possible association between the increased availability of paracetamol in non-pharmacy outlets and the number of reported paracetamol-related intoxications led to the decision to restrict the sale of paracetamol tablets to pharmacies from November 2015 [8]. Diclofenac has also been the subject of controversy, due to its negative environmental impact and the risk of cardiovascular disease. After a comprehensive Danish study concluded that diclofenac poses a significant cardiovascular health risk compared with non-use and use of paracetamol, ibuprofen and naproxen [9], oral formulations of diclofenac were restricted to prescription use only from June 1, 2020.

In Sweden, analgesic drugs are eligible for reimbursement if prescribed for the relief of chronic pain. Hence, most OTC analgesics are used for non-chronic conditions or as supplement to prescribed analgesics. Although each dispensing of OTC analgesics is confined to maximum pack sizes (e.g. 10 g of paracetamol), there are no restrictions on the number of packages of OTC analgesic allowed sold per person per day. Direct-to-consumer advertisement is allowed for OTC drugs under the condition that it provides a current, factual and balanced presentation that promotes appropriate use of the drug.

There are no valid patents for any of the OTC analgesics sold on the Swedish market. Hence, the available products are generics with a given brand name or just a generic name followed by the company name. Although most generic drug policies, which comprise measures to make generic drugs more available, have been directed towards the market for prescription drugs [10], an increase in the number of available generics in the OTC market has also been observed. For instance, in Sweden, up to four new brands of the top-selling analgesics (i.e. paracetamol and ibuprofen) were introduced per substance between 2009 and 2013, all with a lower price compared with the previously established brands [11].

Sweden has had mandatory generic substitution of reimbursed prescription drugs since 2002. Although being mandatory, physicians and pharmacists are allowed to make exceptions due to medical or pharmaceutical reasons. In addition, the patients have the possibility to decline by paying the difference between the price of the prescribed drug and the maximum reimbursement price set by the Dental and Pharmaceutical Benefits Agency [12]. According to the dispensing register, the use of generic drugs as a share of total sales are high, with acceptance rates of generic substitution close to 100% even in therapeutic areas where generic substitution has been considered controversial [13, 14]. Despite this, studies have shown a dissatisfaction with generic drugs in a sizeable minority of Swedish patients [15, 16]. In one of the studies, almost one third reported a low level of trust in the equality of interchangeable generics, of whom 53% still accepted substitution [16]. This apparent contradiction may be due to demand-side measures that stimulate the prescription and/or dispensing of generic drugs. Since most OTC drugs are usually excluded from pharmaceutical benefit schemes, as is the case in Sweden, it is reasonable to expect a demand for cheaper generic products in this segment of the pharmaceutical market. Nevertheless, paradoxically, the brand-name drugs continue to be market leaders despite price being the single most influential factor for consumer choice [17, 18].

The use of generic drugs has been extensively studied with respect to consumer experiences and attitudes (e.g. summarised in several reviews [19–21]). However, just a few studies have focused on OTC generics [17, 18, 22]. One example is Fraeyman et al., who investigated Belgian consumer perceptions of different brands and packages of OTC paracetamol [22]. They found that almost half of the respondents were unable to state an actual brand name of paracetamol and that the majority, with an overrepresentation of lower-educated respondents, believed that there were differences in effects and side effects between the different brands [22].

With the increasing number of generic products available on the market, and the possibility of buying OTC drugs from non-pharmacy outlets, it is crucial with regard to appropriate use that the consumers possess the necessary knowledge to differentiate between the different brands and choose the proper drug for their ailments. The aim of this study was therefore to investigate Swedish consumers’ knowledge of and attitudes towards generic OTC analgesics.

## Material and method

### Material

The study population consisted of 209 Swedish adults (≥ 18 years of age) who bought OTC analgesics at a community pharmacy in either Gothenburg, Malmö or Stockholm, the three largest cities in Sweden. The OTC analgesics available on the Swedish market at the time of data collection contained the following active substances: acetylsalicylic acid, diclofenac, ibuprofen or paracetamol.

### Method

Based on convenience sampling, three community pharmacy managers, one in each of the three cities, were given information about the study before they gave their permission to recruit participants among their customers. Customers who purchased an OTC analgesic were then given oral and written information about the study and invited to take part. Of those who were asked to participate, 49% agreed.

Data were collected in April 2016 (Gothenburg and Stockholm, by MW) and March 2017 (Malmö, by DA) by using a structured questionnaire that the participants completed on site. The questionnaire included items regarding current use of OTC analgesics, knowledge of the active substances in different products, choice of OTC analgesic (generic or original brand), information received about OTC analgesics and experience with generic substitution of prescription drugs. Concerning knowledge, the participants were asked to assign the generic name of four active substances to six different brands of OTC analgesics. Finally, a set of six statements about the participants’ attitudes towards generic drugs were to be answered on a 5-point Likert scale. Information on participants’ sex, age and education was also collected. Three age groups were constructed: 18–39 years, 40–59 years and > 60 years. Education was categorised as “lower education” (ranging from elementary school to postgraduate studies without a degree) and “higher education” (a university degree after a minimum of 3 years of studies at a university level). The questionnaire was piloted in advance.

SPSS Statistics, version 22 (SPSS Inc., Chicago, IL, USA), was used in the analyses of the data. Pearson’s chi-square tests were conducted to examine the association between the participants’ knowledge of the active substances of OTC analgesics and the variables sex, age, educational level, frequency of OTC analgesic use, if they had been offered generic substitution of prescription drugs and choice of OTC analgesic. Logistic regression was used to investigate the odds ratios (ORs) for choosing a generic OTC analgesic in relation to sex, age, educational level, frequency of OTC analgesic use and been offered generic substitution of prescription drugs. Female sex, age 18–39 years, lower education, weekly use of OTC analgesics and not been offered generic substitution of prescription drugs were chosen as reference categories in the analyses. The results are presented as ORs with 95% confidence intervals (CIs).

## Results

The study population consisted of 138 women (66.0%) and 71 men (34.0%) 20–82 years of age (mean age 43.1 years). Of these, 116 participants (55.5%) were categorised as having lower education and 93 participants (44.5%) as having higher education. Twenty-six participants (12.4%) were students, 154 (73.7%) were employed and 29 (13.8%) were retired or receiving government benefits.

Almost one in five (18.2%) reported weekly use of OTC analgesics, while monthly or less than monthly use was reported by 40.7% and 41.1%, respectively. Sixty per cent of the participants reported that they had gotten information from pharmacy personnel about the availability of cheaper brands of OTC analgesics, and 62.2% had experienced being offered generic substitution of prescription drugs.

Table 1 shows how the participants matched the active substances (generic names) to the corresponding brand names. Sixty-seven participants (32.1%) assigned three or four active substances to the correct brand(s) of OTC analgesics on the Swedish market (Table 2). Fifty participants (23.9%), comprising 41 (29.7%) women and 9 (12.7%) men, assigned all four active substances correctly. A majority of these participants (62.0%) had higher education. Eighty per cent of those who gave correct answers had been offered a generic substitute of a prescription drug, and 86.0% had bought generic OTC analgesics.

**Table 1:** The numbers and percentages of participants’ who indicated acetylsalicylic acid, diclofenac, ibuprofen and paracetamol as the active substances in the respective brands of OTC analgesics (*n* = 209). Correct identifications are reported in bold

		Active substance (generic name)			
		Acetylsalicylic acid	Diclofenac^a^	Ibuprofen	Paracetamol
Brand name	Alvedon®	5 (2.4%)	6 (2.9%)	11 (5.3%)	**143 (68.4%)**
	Ibumetin®	14 (6.7%)	6 (2.9%)	**134 (64.1%)**	5 (2.4%)
	Ipren®	7 (3.3%)	6 (2.9%)	**119 (56.9%)**	32 (15.3%)
	Panodil®	11 (5.3%)	3 (1.4%)	13 (6.2%)	**121 (57.9%)**
	Treo®	**141 (67.5%)**	8 (3.8%)	3 (1.4%)	6 (2.9%)
	Voltaren®	8 (3.8%)	**123 (58.9%)**	6 (2.9%)	10 (4.8%)

^a^OTC status for oral formulations was withdrawn from June 1, 2020

**Table 2:** Associations between the numbers of correct matches of brand names with active substances in relation to different factors (*n* = 209)

	0–2 matches	3–4 matches	*p* value*
Sex			
Female	86 (62.3%)	52 (37.7%)	**0.015**
Male	56 (78.9%)	15 (21.1%)	
Age group			
18–39 years	68 (64.2%)	38 (35.8%)	0.327
40–59 years	41 (68.3%)	19 (31.7%)	
≥ 60 years	33 (76.7%)	10 (23.3%)	
Educational level			
Lower	88 (62.0%)	54 (38.0%)	**0.006**
Higher	28 (41.8%)	39 (58.2%)	
Frequency of OTC analgesic use			
At least weekly	27 (71.1%)	11 (28.9%)	0.650
Less than weekly	115 (67.3%)	56 (32.7%)	
Been offered generic substitution of prescription drugs			
No	59 (77.6%)	17 (22.4%)	**0.023**
Yes	83 (62.4%)	50 (37.6%)	
Choice of OTC analgesic			
Generic brand	92 (63.4%)	53 (36.6%)	**0.036**
Original brand	50 (78.1%)	14 (21.9%)	

*Pearson’s chi-square test, *p* < 0.05

As seen in Table 1, the most common mistake was to assign paracetamol as the active substance in products with ibuprofen (17.6%) and vice versa (11.5%).

More than two thirds (69.4%) reported that they choose the cheaper generic brands over original brands when they buy OTC analgesics. According to Table 3, these were more likely to be women than men. There was no association between the frequency of use and choice of generic or original brand. The likelihood of choosing a cheaper brand was two times higher for participants who had experienced being offered a generic substitute of a prescription drug compared with those who had not. A majority of participants reported positive attitudes towards generic OTC analgesics, as can be seen in Table 4.

**Table 3:** Adjusted odds ratios (ORs) for choosing a generic over-the-counter (OTC) analgesic (*n* = 145) over an original brand (*n* = 64), in relation to sex, age, educational level, frequency of OTC analgesic use and experience with generic substitution of prescription drugs

	Choosing a generic over-the counter (OTC) analgesic over an original brand			
	*n*	%	OR	95% CI
Sex				
Female	104	75.4	1.0	
Male	41	57.7	**0.5**	**0.26–0.94**
Age group				
18–39 years	73	68.9	1.0	
40–59 years	43	71.7	1.0	0.46–1.99
≥ 60 years	29	67.4	0.8	0.34–1.78
Educational level				
Lower	76	65.5	1.0	
Higher	69	74.2	1.4	0.75–2.64
Frequency of OTC analgesic use				
At least weekly	28	73.7	1.0	
Less than weekly	117	68.4	0.6	0.27–1.42
Been offered generic substitution of prescription drugs				
No	45	59.2	1.0	
Yes	100	75.2	**2.0**	**1.03–3.87**

**Table 4:** Participants’ attitudes towards generic OTC analgesics (*n* = 208)

Statement	Corresponds very well, *n* (%)	Corresponds fairly well, *n* (%)	Corresponds neither well nor poorly, *n* (%)	Corresponds fairly poorly, *n* (%)	Corresponds very poorly, *n* (%)
1. I am positive towards cheaper brands on the market	122 (58.7%)	47 (22.6%)	21 (10.1%)	10 (4.8%)	8 (3.8%)
2. I feel safe when I buy cheaper brands	69 (33.2%)	59 (28.4%)	52 (25.0%)	18 (8.7%)	10 (4.8%)
3. A lower drug price entails poorer quality	2 (1.0%)	17 (8.2%)	58 (27.9%)	52 (25.0%)	79 (38.0%)
4. Cheaper brands are of the same quality as the more expensive brands	56 (26.9%)	51 (24.5%)	63 (30.3%)	27 (13.0%)	11 (5.3%)

## Discussion

This study shows that Swedish consumers, who were recruited during a visit to the pharmacy, to a varying extent have the knowledge needed to identify the active substances in commonly used OTC analgesics. One third of the participants matched at least three out of four active substances to the correct brand. The shares of correct identification varied from 57% for one of the ibuprofen products to 68% for the acetylsalicylic acid product and one of the paracetamol products. In comparison, 46% of the consumers in the Belgian study were able to identify brand names for paracetamol [22]. In an American study, 26% of the paracetamol users reported knowledge of the active substance in paracetamol-containing products [18].

Two thirds of the participants in this study were women, which reflects that there are more women than men among pharmacy customers and that women use OTC drugs more frequently compared with men [3, 23, 24]. Regarding analgesics, this may be related to menstrual pain and that women seem to suffer from pain at rates higher than men and are more sensitive to pain [25]. It was found that a higher proportion of women than men could identify the active substances of the OTC analgesics (37.7 vs. 21.1%, Table 2). Since women have more frequent visits to the pharmacy, they may also receive more information about generic drugs and drugs in general. A previous Swedish study showed that women to a larger extent than men report pharmacy staff as a source of information [24]. In the same study, men, young adults (18–39 years) and individuals with low education were identified as the most frequent buyers of OTC drugs in non-pharmacy outlets, when taking into account the total number of purchases [24]. This may further explain the discrepancy between the sexes, since the non-professional personnel are not supposed to provide information upon sale. As in previous research, better knowledge turned out to increase proportionally with higher education [26].

Almost one third reported that they would choose the original brands over cheaper generics when they buy OTC analgesics. Participants who were male and/or had never been offered generic substitution of a prescription drug were most unlikely to choose a generic brand over an original. There was also an association between a negative attitude towards generics and being able to match a maximum of two active substances with the correct brand. The results on attitudes in this study are in line with results from studies on generic prescription drugs [16, 19–21]. A systematic review of studies from a wide range of countries showed that about one third of the general population hold negative views of generic drugs [19], a finding which coincides with a review restricted to studies from high-income countries [20] and the before-mentioned Swedish study [16]. Possible explanations for the negative attitudes towards generics are the common lay belief that lower prices imply poorer quality and the familiarity and trust people tend to have in well-known brands [21]. The previously cited Belgian study showed a shift in preference—from the more expensive original brand to the cheaper generics—for two thirds of the consumers when the prices were displayed, whereas the remaining third stayed loyal to their brands despite the price difference [22]. This brand loyalty was especially seen in lower-educated consumers, who believed that effectiveness and quality differed between brands, and among older consumers, who wanted to avoid confusion because of different packages [22]. Studies have also shown that patients make trade-offs between price and perceived quality in relation to the seriousness and complexity of the condition they are treating, when choosing among original and generic brands [27–29]. This again can be explained by a lack of understanding of different products having the same active ingredients.

Previous studies have concluded that Swedes report responsible use and basic awareness of paracetamol regarding the maximum daily dose and potential consequences of overdosing [24, 26]. Paracetamol may, however, present a special case, since this substance received a lot of media attention in 2015 after its sale was withdrawn from non-pharmacy outlets [30]. On the other hand, official statistics in Sweden show that this act was followed by an increase in the defined daily doses of ibuprofen sold outside pharmacies that equalled the respective decrease in the sale of paracetamol [31]. This suggests that individuals are either unaware or careless about when it is appropriate to use one drug instead of the other. It may also be that they do not know that the products contain different active ingredients, something that is substantiated by the results of this study. Another aspect of this is the risk of erroneous use. A study from Finland showed that 26% of all problems related to OTC drugs concerned so-called high-risk drugs, in which many of these cases involved drug-drug interaction between NSAIDs and prescription drugs and inappropriate duplication of NSAIDs [32]. Unintentional duplication of active ingredients due to misunderstanding has also been seen when making inventories of chronic patients’ drug use [33, 34].

In general, familiarity with the name or brand, in addition to the perceived effectiveness and safety of the drug, is known to influence consumers when buying OTC drugs [35]. Another influencing factor that cannot be ignored is direct-to-consumer advertising [17]. The salient point is whether consumers have the necessary knowledge to differentiate between the different brands and choose the appropriate drug for their ailments in situations where they do not receive counselling. Now that a range of products is being sold outside of regular pharmacies, it is important to increase the knowledge of OTC analgesics. Awareness of the name of the active substances should be raised, for instance, by highlighting the generic name on the drug packages, to reduce any confusion and mistakes that may compromise the treatment outcome and cause unintended effects. Regarding increasing consumers’ knowledge of the OTC analgesics’ risk profiles, previous studies have shown that written information in itself does not yield any significant results [36, 37], nor does the counselling from pharmacy personnel [36], while counselling by physicians is associated with better results [36]. Schmitt et al. have suggested that a better coordination of physician and pharmacist counselling, integrated with written information, may improve efficiency and eliminate redundancy to communicate clear and complete information [36]. That physicians discuss the use of OTC drugs with chronic patients is advantageous, since they also have insight into patients’ use of prescription drugs. To benefit individuals who do not regularly visit a physician, a combination of better labelling of OTC drugs and more active counselling by pharmacy personnel should be a goal.

This study was conducted in the three largest cities in Sweden, where there are high numbers of pharmacies. Possibly, living in a big city with many pharmacies increases people’s knowledge of OTC drugs. The fact that we only recruited pharmacy customers may also be a reason for a possible overestimation of people’s knowledge. Yet, we consider this a modest bias, since a majority of Swedes still buy most of their OTC drugs at a pharmacy [3, 24]. Furthermore, patient information leaflets are reportedly the consumers’ most common source of information [24]. The response rate of about 50% is a limitation of the study. There is always a risk that those with the greatest interest in and knowledge of the topic agree to participate. There was also a high proportion of people with a university degree (45%) compared with the national level (28%), which indicates that the results of the present study overestimate the extent of knowledge of OTC analgesics in the country as a whole. However, it corresponds very well with the educational level in the largest cities where the study was conducted [38].

## Conclusion

This study shows that Swedish pharmacy customers to a varying extent have the knowledge needed to identify the active substances in commonly used OTC analgesics. Knowledge of the names of active substances of different brands was associated with positive attitudes towards generic products. There seems to be a predominantly positive attitude towards generic OTC analgesics among Swedish consumers, although some consider generic drugs to be of inferior quality and stay loyal to the brand-name drugs.

Raised awareness of the names of the active substances of OTC analgesics is important, alongside increased knowledge of the drugs’ similarities and differences in effects and risk profiles. Better labelling of the packages and more counselling about OTC analgesics from different sources seem necessary for the consumers to be able to differentiate between the different brands and choose the appropriate drugs for their ailments.
